# Competencies for Global Mental Health: Developing Training Objectives for a Post-Graduate Fellowship for Psychiatrists

**DOI:** 10.29024/aogh.2382

**Published:** 2018-11-05

**Authors:** Colin Buzza, Anna Fiskin, Jorien Campbell, Jennifer Guo, Jacob Izenberg, Barbara Kamholz, Erick Hung, Bibhav Acharya

**Affiliations:** 1 Department of Psychiatry, University of California, San Francisco, San Francisco, CA, US; 2 San Francisco Veterans Affairs Medical Center, San Francisco, CA, US; 3 Possible, a non-profit organization, Achham, NP

## Abstract

**Background:**

Despite an increase in psychiatry trainees’ interest in global mental health (GMH), there is a lack of relevant training competencies developed using educational frameworks that incorporate viewpoints from high- and low-income countries.

**Objective:**

The aim of this study was to determine competencies for a two-year post-graduate GMH fellowship for psychiatrists utilizing Kern’s six-step process as a theoretical framework for curriculum development.

**Methods:**

We conducted a targeted needs assessment via key informant interviews with a purposive sample of stakeholders (n = 19), including psychiatry trainees, generalist clinicians, medical directors, psychiatrists, researchers, and GMH educators from high- and low-resource settings in the United States and abroad. We analyzed data using a template method of thematic analysis.

**Findings:**

We tabulated learning objectives across 20 domains. Broadly, clinical objectives focused on providing supervision for short-term, evidence-based psychotherapies and on identifying red flags and avoiding harmful medication use among vulnerable populations such as children and the elderly. Non-clinical objectives focused on social determinants of health, education, and clinical supervision as part of capacity-building for non-specialists, engagement in a systems-wide project to improve care, and ethical and equitable partnerships that involve reciprocal and bidirectional education. Several competencies were also relevant for global health work in general.

**Conclusions:**

A theory-informed framework for curriculum development and a diverse set of key informants can provide educational objectives that meet the priorities of the trainees and the clinical sites in both low- and high-income settings. Limitations of this study include a small sample size and a focus on clinical needs of specific sites, both of which may affect generalizability. Given the focus on training specialists (psychiatrists), the low-resource sites highlighted the importance of educating and supervising their permanent, generalist clinicians, rather than providing direct, independent patient care.

## Introduction

With growing recognition of challenges posed by the worldwide burden of mental illness and the scarcity of mental health care in many low-resource settings, global mental health (GMH) has been increasingly identified as a training priority [1,2]. There have been some efforts to describe a framework for global health education in general [3,4,5]. GMH competencies have been described for non-specialists in low-resource settings [6,7]. However, the delivery of GMH training has been limited by few training opportunities and a lack of data-driven training objectives for clinical specialists such as psychiatrists.

Only a minority of US psychiatric residency programs offer formal opportunities in GMH education [8]. Dedicated post-graduate GMH training programs are rarer. Prior to the development of the fellowship program described here, there were only four US-based GMH fellowships, three of which focused exclusively on research [9,10,11,12,13]. Some of these research fellowships [9,10] and a graduate-level GMH program [14] have disseminated descriptions of their general structure and approach. However, a structured strategy to develop relevant competencies, learning objectives, and educational strategies for GMH training for post-graduate psychiatrists has not been described in the US or elsewhere.

Competency development is often based on expert opinion of faculty members in high-resource settings and is not driven by conceptual frameworks [15]. This could lead to objectives that may not match the needs of the intended beneficiaries. We describe steps utilized in developing a curriculum for the University of California San Francisco (UCSF) Psychiatry HEAL Fellowship in Global Mental Health, a two-year post-residency program for psychiatrists. We discuss the process and outcomes of a needs assessment that informed the development of learning objectives and educational strategies for the fellowship.

## Methods

### Theoretical framework

Our group utilized Kern’s Six-Step Model of Curriculum Development [16], which provides an iterative framework to develop, implement, and evaluate a medical curriculum by following the sequential steps of (1) problem identification and general needs assessment, (2) targeted needs assessment, (3) development of goals and objectives (e.g., competencies and specific learning objectives), (4) design of educational strategies, (5) curriculum implementation, and (6) evaluation and feedback. The first four steps of our curriculum development process are reported here.

### Curriculum Development Team

A group was assembled from the UCSF Department of Psychiatry under leadership of the fellowship director (BA). Membership consisted of residents (JB, CB, JG, JI), a fellow (AF), and faculty (BA, BK). Members had diverse backgrounds including experiences in global health and GMH, public health, social sciences, curriculum development, teaching, and research. The group met every one to three months from December 2014 to May 2016, with additional meetings among working groups formed within team.

### Problem Identification and General Needs Assessment

Based on Kern’s model, the group first identified the broader problem it was trying to address through consensus based on intensive discussions informed by expertise and prior experience. Subsequently, a literature review was conducted to inform a general needs assessment based on MEDLINE search of peer-reviewed literature of keywords “Global Mental Health” and “training” or “education” or “psychiatrist” or “fellowship” or “residency” or “competencies”.

### Targeted Needs Assessment

For the second step of Kern’s model, the group was divided into two parallel teams. A “fellows” team assessed the gap between competencies needed to be an independent GMH practitioner and those available at the end of a US-based residency training in general psychiatry. A “site” team assessed the gap between the optimal human and other resources needed to deliver high-quality mental health care and the existing infrastructure of the site, and how a GMH practitioner could assist in bridging that gap. The teams identified key informants (N = 19) using a purposive method [17]. The fellows team interviewed directors of the existing US GMH fellowships (n = 4), directors of other graduate GMH programs (n = 3), psychiatrists who are current or former GMH trainees (n = 3), and psychiatry residents with a strong interest in GMH who self-identified as potential fellowship applicants (n = 3). The site team interviewed a psychiatrist and the medical director from each of the two sites of the fellowship program: an IHS site in the US and a district-level hospital in Nepal (n = 4). In addition, the site team interviewed generalist physicians at two sites based in low-income countries that have previously hosted GMH trainees and do not have on-site psychiatrists (n = 2).

The interviews were conducted between February and May 2016. Interviews were each between 30 and 60 minutes in length and conducted either in person, via phone, or via teleconferencing. Interviews were conducted using semi-structured interview tools developed by each team to assess the specific gap identified in the previous step. The fellows team interviews focused on the experiences of those who have provided or received GMH training as well as the goals of those who are seeking GMH training. Interview themes included: clinical, research, and administrative skills; mentorship and support; and career development. The site team interviews focused on the needs, capacity, and goals of training sites and experiences of those who have previously hosted fellows. Themes included: clinical needs and priorities; clinical resources and infrastructure; potential roles of fellow; opportunities for mentorship and support; and key structural and cultural considerations. Interview data was captured using structured notes and then collated into a master document. Utilizing a template method, data were coded by BA using thematic analysis and the results were shared with the larger working group, which further refined the codes. This iterative process was continued until consensus was achieved. A summary of interview data is available in Supplement 1.

### Development of Goals and Objectives

The working group reviewed the products of the general and targeted needs assessments and converted the emerging themes into specific competencies nested within competency domains. These were reviewed by the group in multiple iterations, with rounds of discussion and refinement until consensus was achieved. Content experts were identified for each domain among the faculty of UCSF Department of Psychiatry. The working group members and the content experts worked in partnership to develop specific, measurable learning objectives for each competency [16].

## Results

### Problem Identification

The team identified two intertwined problems based on their prior experience and pre-study, informal discussions with leaders at the host sites: 1) the need to improve and expand post-graduate medical training in GMH, with a particular focus on clinical training, education, and systems improvement; and 2) the need to improve mental health care and build capacity for addressing mental illness and substance use at host sites.

### General Needs Assessment

The results of the literature review to understand general issues in GMH training included publications examining existing GMH training [8,18], advocating for increased GMH research training [10] and improved GMH clinical training [19,20], and discussing ethics in GMH training [21]. The search also yielded descriptions of one international GMH educational partnership [22], one graduate-level GMH training program [14], and two GMH research fellowships [9,10]. However, there were limited online or published curricular information from training programs in GMH and no published curricula from existing post-graduate clinical GMH fellowships.

### Developing Goals and Objectives from Targeted Needs Assessment

This step resulted in numerous themes that are nested within 20 competency domains, as listed in Table 1. Many competencies were specific to GMH and several were also relevant to the general practice of global health.

**Table 1:** Integrated competencies for global mental health fellowship.

	Domain	Competencies

**1.**	**Structural Determinants of Mental Health**	*Goal: Describe the impact of structural determinants on health* A. Define structural competence and describe its key components ^GMH^ B. Describe common social, historical, structural, political, and economic determinants of mental health ^GMH^ C. Describe and compare key social, historical, structural, political, and economic determinants of mental health within different local contexts ^GMH^ D. Explain the historical and current role of global health actors, including types of global health partnerships and programs ^GH^ E. Demonstrate in-depth knowledge of diseases that disproportionately affect the poor ^GH^
**2.**	**Cultural Aspects of Mental Health**	*Goal: Deliver mental healthcare in cross-cultural settings with cultural humility* A. Describe and critique traditional models of cultural competency in mental health ^GMH^ B. Develop and utilize strategies to appreciate and continually refine an understanding of cultural influences on mental health and illness ^GMH^ C. Describe and compare locally-specific traditional healing practices ^GMH^ D. Understand how to incorporate traditional healing practices into clinical care ^GMH^
**3.**	**Understanding the Health System & Resources in Mental Health**	*Goal: Gain fluency in systems-level approach to improving access to mental health services* A. Explain the importance of health systems strengthening and health systems redesign to improving health outcomes ^GH^ B. Illustrate and contrast the roles of health systems design in either reinforcing health inequities or helping overcome them ^GH^ C. Analyze the impact of several common approaches to health care financing on health care access, health outcomes, and health equity ^GH^ D. Analyze the health system of domestic and international sites to determine: leadership, chain of command for decision making, funding streams, supply chain, interaction with local community, and players in the health system ^GH^ E. Describe and compare mental health and related resources available within specific countries and regions ^GMH^ F. Analyze and compare mental health and related resources available in local communities ^GMH^ G. Analyze and compare mental health and related resources available at local clinical sites ^GMH^ H. Describe factors that affect access to mental health and related resources at the aforementioned levels ^GMH^
**4.**	**Engagement in the Health System**	*Goal: Gain experiential knowledge in strengthening health care delivery systems* A. Develop skills to design, implement, monitor and/or evaluate health programs and health systems, including their inputs, outputs, effectiveness, cost-effectiveness, and financial management ^GH^ B. Elicit the goals of the local health system in choosing and implementing your projects ^GH^ C. Engage with local leaders/civic society groups targeting other social determinants of health including access to clean water, housing, food security, and economic opportunities ^GH^ D. Develop a project/intervention in partnership with local partners at your site(s) that aims to improve measurable disease-specific outcomes through health-systems level change ^GH^
**5.**	**General Provision of Care**	*Goal: Gain experiential knowledge in delivering health care in a low-resource settings* A. Determine the local burden of communicable and non-communicable diseases within your setting ^GH^ B. Apply the evidence-based international standards of care for the diseases affecting your population ^GH^ C. Demonstrate skills in adapting to a resource-limited setting while maintaining a high quality standard ^GH^ D. Target interventions to address both prevention and management of the most common disease in the local context ^GH^ E. Demonstrate competency in basic medical procedures, including bedside ultrasound, phlebotomy, and selected lab medicine techniques if relevant to your profession ^GH^ F. Demonstrate strong, culturally appropriate communication skills in interprofessional and patient interactions ^GH^
**6.**	**Emergency Psychiatry and Inpatient Care**	*Goal: Gain clinical competence in delivering care for severe and emergent mental health problems* A. Describe the most common causes of acute psychiatric presentation in different locales and formulate differential diagnoses that are site- and culture-specific ^GMH^ B. Formulate risk assessments that take into account local medical and cultural factors ^GMH^ C. Identify local laws and practices that apply in psychiatric emergencies and inpatient care ^GMH^ D. Describe systemic challenges specific to delivery of emergency and inpatient psychiatric care in low resource settings ^GMH^ E. Describe the local availability, indications, contraindications, and goals for inpatient psychiatric hospitalization ^GMH^ F. Identify the local availability and forms of medication used in different clinical settings and know how to use and monitor them appropriately in each setting ^GMH^
**7.**	**Adult Community Psychiatry**	*Goal: Gain clinical competency in delivering care for common mental illnesses for adults by utilizing and improving local resources* A. Describe common presentations, differential diagnoses, and appropriate interventions for each major mental illness in local community settings ^GMH^ B. Identify the availability of psychotropic medications of each major class in different community settings, and know how to use and monitor them appropriately in each ^GMH^ C. Describe systemic challenges specific to delivery of outpatient and community mental health care in different low resource settings ^GMH^ D. Identify key non-professional providers of mental health care and psychosocial support and demonstrate locally-appropriate task sharing ^GMH^
**8.**	**Child and Adolescent Psychiatry**	*Goal: Recognize signs of pathology, avoid harm, and deliver basic care based on specialist recommendation to assist children* A. Make comparisons between cultural perspectives on child development ^GMH^ B. Describe local and culturally-specific parenting strategies to promote positive child behavior ^GMH^ C. Describe local and culturally-specific attributions of child mental health ^GMH^ D. Develop and apply culturally-appropriate behavioral plans that encourage healthy behaviors ^GMH^ E. Identify local and culturally-specific risk assessments and interventions for suicide and self-harm in children and adolescents ^GMH^ F. Describe site- and culture-specific common presentations, differential diagnoses, and prognoses of psychiatric disorders that first present in childhood ^GMH^ G. Describe site- and culture-specific common presentations, differential diagnoses, and prognoses of children and adolescents with early onset of adult psychiatric disorders ^GMH^ H. Identify the availability and demonstrate locally-appropriate use of both pharmacologic and non-pharmacologic treatments for psychiatric disorders in children and adolescents ^GMH^ I. Describe local considerations for safe prescribing of medication to children and adolescents ^GMH^
**9.**	**Geriatric Psychiatry**	*Goal: Recognize signs of pathology, avoid harm, and deliver basic care based on specialist recommendation to assist children* A. Describe site- and culture-specific common presentations, differential diagnoses, and management of common mental illnesses such as depression and anxiety within geriatric populations ^GMH^ B. Describe the local and culturally-specific impact of geriatric depression and other mental illness on family structure and function ^GMH^ C. Describe local and culturally--specific conceptualizations of dementia and cognitive impairment ^GMH^ D. Describe the local and culturally-specific impact of dementia and cognitive impairment on family structure and function ^GMH^ E. Demonstrate how to measure cognition in a locally appropriate and acceptable way ^GMH^ F. Identify and utilize locally available and appropriate pharmacologic and non-pharmacologic interventions for cognitive impairment and dementia ^GMH^ G. Identify locally-specific risks for delirium and describe how to best address it with the resources available ^GMH^ H. Demonstrate the ability to talk to patients and families about cognitive impairment, dementia, and delirium in a culturally-appropriate manner ^GMH^
**10.**	**HIV Psychiatry**	*Goal: Deliver mental health care for people with HIV with critical awareness of specific issues in this population* A. Identify the local breadth and prevalence of neuropsychiatric disorders amongst people living with HIV ^GMH^ B. Describe neuropsychiatric symptoms associated with HIV infection and understand common site- and culture-specific presentations and differential diagnoses ^GMH^ C. Explain evidence-based harm-reduction strategies to address substance use and reduce transmission among people with HIV, and identify locally appropriate utilization of such strategies ^GMH^ D. Describe common interactions between HIV and psychiatric medications and develop a rational, site- and culture-specific approach to prescribing psychiatric medications for people living with HIV ^GMH^ E. Identify common psychiatric side effects of locally-available HIV medications ^GMH^ F. Describe the site- and culture-specific impact of seroconversion, stigma, and discrimination for those living with HIV ^GMH^
**11.**	**Psychotherapy**	*Goal: Strengthen local capacity to deliver evidence-based psychotherapies* A. Describe the key components of and indications for evidence-based psychotherapies, including major cross-cultural evidence and adaptations ^GMH^ B. Identify locally-available resources for psychotherapy ^GMH^ C. Develop locally specific thresholds for therapy referrals ^GMH^ D. Provide locally-appropriate guidance and supervision for other health workers implementing psychotherapy for patients ^GMH^
**12.**	**Substance Use**	*Goal: Recognize and address common sources of substance use* A. List locally-common substances of abuse ^GMH^ B. Describe local demographic patterns associated with high-burden substances ^GMH^ C. Describe locally-specific societal impacts of substance abuse ^GMH^ D. List locally-available treatments, both in the community and the hospital, for acute and chronic substance use problems ^GMH^ E. Provide other health workers with locally-appropriate training and supervision in recognizing and addressing common substance use problems ^GMH^
**13.**	**Training and Education**	*Goal: Utilize training and education to enhance local capacity in improving mental health care* A. Recognize the importance of learning climate, goal setting, knowledge retention, evaluation and feedback in effective teaching ^GH^ B. Describe techniques for effective cross-cultural teaching ^GH^ C. Gain comfort in multiple arenas of teaching, including one-on-one teaching, small and large group settings ^GH^. D. Describe and apply strategies of evaluation for your trainees, peers, and mentors ^GH^ E. Develop independent goals and objectives tailored to your personal strengths and weaknesses ^GH^ F. Develop effective strategies to be both a mentee and a mentor ^GH^
**14.**	**Clinical Supervision**	*Goal: Strengthen systems to provide ongoing supervision for non-specialists in delivering high-quality mental health care* A. Describe the collaborative care model and describe efforts to adapt it to different cultures and settings ^GMH^ B. Develop a clinical registry to review all patients receiving mental health services ^GMH^ C. Develop a locally-appropriate structure to meet with generalist clinicians to review difficult cases and provide clinical support ^GMH^ D. Develop a locally-appropriate structure to provide urgent or emergent support for generalist clinicians ^GMH^ E. Develop a locally-appropriate evaluation plan to assess the effectiveness of clinical supervision for clinicians and patient outcomes ^GMH^ F. Identify common and serious clinical issues presented during registry review and supervision and provide related training for all clinicians ^GMH^
**15.**	**Interprofessionalism and Leadership**	*Goal: Enhance your own leadership and collaborative skills* A. Recognize your leadership style and that of your colleagues ^GH^ B. Apply skills of management, including delegation, moderation, supervision, and evaluation within your project teams ^GH^ C. Be able to articulate a vision to others in the field ^GH^ D. Describe conflict resolution styles ^GH^ E. Develop and apply the communication skills to advance and disseminate your work in a public arena ^GH^ F. Describe and practice applying strategies of leading without authority ^GH^ G. Describe the model of clinical accompaniment and use this model in your clinical setting ^GH^ H. Model professionalism in your role as a healthcare provider, colleague, and patient advocate ^GH^ I. Create an interprofessional collaborative environment among local and international nurses, physicians, and other health care professionals ^GH^
**16.**	**Advocacy**	*Goal: Enhance your own skills in advocating for people living with and at risk for mental illness* A. Identify types of advocacy and the methods that they employ to advance a particular cause ^GH^ B. Identify key actors, audiences, policies, and policy makers within your area of advocacy ^GH^ C. Raise awareness among local population and healthcare professionals on specific disease conditions or prevention efforts ^GH^ D. Create written or media advocacy pieces with local partners ^GH^ E. Disseminate your work in scientific and academic circles ^GH^ F. Write 1 blog post each quarter to advocate for specific areas of interest ^GH^
**17.**	**Health Equity & Ethics**	*Goal: Gain theoretical and experiential knowledge in ethical practice of global health to address inequity* A. Analyze your health system from an equity perspective ^GH^ B. Integrate principles of social medicine into your approach to global health delivery ^GH^ C. Demonstrate increased recognition of ethical issues involved in global health work ^GH^ D. Identify strategies for dealing with these ethical issues as they arise ^GH^ E. Ensure the ethical and responsible conduct in the design, implementation and dissemination of global health research or quality improvement ^GH^ F. Involve your local colleagues in every aspect of program or project development, including any publications or presentations ^GH^ G. Describe the responsibilities of academic centers and US based clinicians in adequately preparing for global health work in communities that are not their own ^GH^ H. Establish regular and transparent communication with your colleagues, in which partners are encouraged to voice their own objectives, challenges, and feedback ^GH^
**18.**	**Self-Reflection and Self-Care**	*Goal: Utilize skills in self-reflection and self-care to maintain long-term engagement in global mental health* A. Describe countertransference and common challenges to countertransference caused by relocation as well as differences in culture and language ^GMH^ B. Be capable of recognizing signs of burnout and its impact on personal health and clinical care ^GMH^ C. Utilize supervision structures to pro-actively identify and discuss emotional reactions to patient care and fellowship responsibilities ^GMH^ D. Utilize supervision structures to pro-actively identify challenges to one’s personal health and ways to maintain physical and mental health ^GMH^
**19.**	**Quality Improvement**	*Goal: Gain experiential knowledge in conducting a quality improvement project* A. Describe the core elements of quality improvement and apply them in the field ^GH^ B. Identify the quality improvement team within your institution. If one does not exist, identify stakeholders in quality improvement ^GH^ C. Partner with the QI team and key stakeholders to choose priorities for quality improvement interventions ^GH^
**20.**	**Research in Global Mental Health (Optional)**	*Goal: Gain basic competence in developing a research protocol in GMH* A. Identify a small, feasible research question relevant to your site ^GMH^ B. Conduct a literature review relevant to your research question ^GMH^ C. Describe types of study design, data analysis and management, sample size calculations, and power calculations ^GMH^ D. Identify the inferences that may be drawn from findings of clinical research studies, including any limitations more common within global mental health research ^GMH^ E. Draft a clinical research protocol for use in the proposed research project ^GMH^ F. Provide peer review and be able to critique research protocols ^GMH^ G. Recognize and address ethical concerns in global mental health research ^GMH^

GH: Competencies for all global health fellowships. GMH: Competencies for the Global Mental Health Fellowship.

Broadly, the competencies can be divided into clinical and non-clinical domains. Under clinical domains, there was specific focus on children and the elderly as vulnerable populations. Given the severe shortage of psychiatrists specialized for these two groups, the GMH practitioner will need to recognize signs of pathology, avoid harm from pharmacological treatments that may not be tolerable to these groups, and to provide care based on recommendations from an off-site specialist. Although providing care for adult patients is the focus on general residency training for psychiatrists, there was a specific focus on utilizing the World Health Organization (WHO) Essential Medications list because psychiatrists trained in high-resource countries may not be familiar with them. HIV psychiatry and substance use were identified as two specific clinical domains given the prevalence and comorbidity with common mental illnesses in low-resource settings. Although general psychiatrists learn some psychotherapies, particularly cognitive and behavioral therapy, the WHO recommends few other techniques that may not be covered in general residency training: group interpersonal psychotherapy, problem management therapy (based on problem-solving therapy), and motivational enhancement therapy. Even if GMH practitioners may not deliver psychotherapy because of linguistic/cultural differences and/or lack of prior training, they may still need to be aware of indications, success rates, and basic components of these therapies when they have a leadership role in mental health care delivery systems.

Non-clinical domains emphasized the role of structural determinants of health and the specific role of culture in mental health. Structural competency [23] is identified as a way to directly engage with stigma, inequity, and non-biomedical determinants that sustain worse outcomes among populations. The role of culture was highlighted in both international work and among the Navajo in the US. It included a critique of traditional models of “cultural competency” and sought to welcome lessons from social sciences to meaningfully improve, rather than curtail, access of non-western populations to mental health services.

Although the themes included several competencies in directly delivering care, there was a strong emphasis on strengthening health systems rather than independently treating patients. This is consistent with the acknowledgement of the importance of task-sharing in GMH [24,25]. Themes of training and education were noted for both global health in general and GMH in particular. In addition, there was acknowledgement of the limited utility of training without ongoing supervision. For task-sharing models, the psychiatrist role of supervision of generalists was emphasized as way to expand access to care while maintaining quality [26]. Furthermore, themes included understanding the health care system and developing a quality improvement project to make systems-level changes to address specific challenges in delivering care. Results that emphasize taking the long view in GMH demonstrate a move away from the “mission” trips that had characterized much of global health work in the past century.

Themes that were unique to the results from the “fellows” team included the importance of self-care to avoid burnout and to become aware of issues of transference and countertransference in a new setting. They also included research work in GMH. This was considered one viable strategy to sustain engagement in GMH, given the lack of dedicated funding streams for GMH practitioners. Upon review of these themes by faculty experts, this specific domain was recommended for an optional competency given the dedicated time necessary to build and execute research projects.

### Educational Strategies

The GMH fellowship experience is provided in partnership between the UCSF Department of Psychiatry and the HEAL Initiative, an existing global health collaborative between UCSF, Indian Health Service (IHS), and numerous institutions and clinical sites in low-resource settings, described in detail elsewhere [27]. In its other fellowships (e.g., global family medicine), HEAL provides global health training for generalist clinicians and includes an intensive two-week “bootcamp,” followed by two years of clinical placements. Post-graduate fellows who have completed residency training rotate between a domestic, under-served site and a low-resource international site. With supervision by local and remote mentors, fellows complete individualized learning plans designed to meet global health competencies developed for the specific fellowship. Learning is supported by a longitudinal curriculum that is administered over the two years, as well as coursework from both the all-fellows “bootcamp” and an online MPH. In addition, the fellowships include reciprocal training where the sites (IHS and international institutions) nominate fellows from among their own staff to receive training from HEAL.

The new GMH fellowship curriculum builds on the existing educational infrastructure of these existing HEAL fellowships, including an intensive, two-week “bootcamp” at the start of fellowship, a shared online platform for content delivery, and fellow placement at domestic (an IHS facility in Navajo Nation) and international sites (*Possible*, a non-profit organization delivery healthcare services in two under-served locations in Nepal). The Department of Psychiatry delivers the longitudinal GMH curriculum described here, as well as provides mentorship for the GMH fellows.

The new GMH curriculum is based on social learning theory, which was most appropriate for this fellowship given the importance of experiential learning in global health delivery. This includes placement in clinical sites with dedicated mentorship and co-placement with fellows from other disciplines to allow social learning. The experiential component is complemented by a longitudinal curriculum delivered over the course of two years during the fellowship. This longitudinal curriculum utilizes both cognitive strategies (e.g., lectures on indications, appropriate use, and adverse effects of WHO essential medications) and experiential strategies (e.g., conducting a mini-ethnographic project to understand local child mental health issues and debriefing on the results with a child psychiatrist). While some educational strategies are delivered locally, many are available remotely (e.g., readings and narrated lectures). Specifically, educational strategies include:

- Live lectures and workshops on GMH provided to all HEAL global health fellows during the bootcamp
- Original narrated lectures custom-developed by faculty experts available remotely and on-demand to GMH fellows
- Biweekly conference calls to cover GMH topics and to engage in discussions with the content expert following the narrated lectures
- Coursework from the formal MPH program
- Readings and written reflections
- Supervision with local and remote mentors
- On-site clinical and educational projects
- An original scholarly project with a specific focus on systems improvement

## Discussion

While some global health curriculum development has been criticized for relatively top-down approach [28,29], our broad needs assessment included significant input from local sites and GMH trainees, in addition to experts and leaders in the field. This allowed development of competencies and educational strategies that balance the needs of learners with the needs of under-resourced training sites. For example, proficiencies such as task-shifting have previously been highlighted as priorities for GMH practitioners [1,2,30], but input from local sites suggested a need for fellows to become proficient in more robust methods of collaborative care. There was particular emphasis on training and supporting other on-site clinicians, who may not have received any prior mental health training [31]. Further, identification of local clinical needs and gaps in local expertise led to competencies in numerous clinical domains, such as emergency psychiatry and HIV psychiatry, that will guide fellows in adapting and building on their existing expertise in new contexts.

Our competency domains allowed us to identify faculty members who were not always GMH experts, but could provide content expertise and educational materials for the specific domains. For example, content for the “training and education” competencies will be delivered by faculty with expertise in education, but limited experience in GMH practice. This strategy may be helpful in academic centers attempting to meet demand for GMH training with limitations on faculty expertise.

In addition to recruiting domain-specific faculty, we employed several strategies to enhance feasibility. We partnered with an existing, general global health program, allowing us to synergize learning and build on existing partnerships. A common challenge in a needs assessment exercise that incorporates multiple viewpoints is that the desired competency list can expand beyond what is feasible. We addressed this by merging competencies with general global health training and also by incorporating domain experts’ opinions on feasibility. For example, the domain on child and adolescent psychiatry was quite extensive but after discussions with faculty, the overarching goal was limited to essential tasks like identifying red flags, avoiding harm, and deliver basic care with close supervision from a specialist. Similar goals were utilized for other sub-specialty domains like geriatric psychiatry and HIV psychiatry, enhancing the overall feasibility of the curriculum.

One factor that we see as strength of our approach, but which may limit its generalizability, is that we built on an existing, university-wide global health program. This provided an existing platform for delivery of general content relevant to all global health practitioners, and allowed us to more clearly focus on developing GMH competencies and training. This strategy has fostered collaboration with other disciplines, and may serve as a template to facilitate development of GMH training programs at other institutions. However, there will be variability in the scope of existing global health programs among institutions interested in replicating our work.

An important limitation of our approach is that generalizability could be affected by the weight placed on local clinical priorities that were identified by a limited number of key clinicians at the two fellowship sites and two additional sites in low-income countries. For example, while the broader GMH priority of collaborative care and capacity-building was emphasized by both clinical sites, other GMH topics such as refugee mental health were prioritized less. While this latter topic will still be included in the curriculum under the umbrella of structural determinants, it may have been more robustly emphasized by other sites not included in the fellowship and needs assessment.

Despite their breadth, the competencies may also inadequately capture some of the relational, self-reflexive, and process-based skills needed to engage with communities and mental health systems in new localities often different from those in which practitioners were raised or trained. This challenge of transferability has been highlighted in broader critiques of utilizing competencies in global health education [29]. Multiple fellowship sites may allow for more synthesis and transferability of process-based skills, though this challenge will require ongoing attention within educational strategies and the design of meaningful assessment methods. Other potential challenges include delivering curricular materials at remote sites and developing structures for local and remote mentorship. We expect to address these limitations in a future publication.

To our knowledge, this is the first publication of competencies developed through a thorough needs assessment to guide a post-graduate GMH curriculum. The process highlighted some of the challenges in developing training for a nascent field where the professional roles are not yet clearly defined and where the needs and relevant skills may vary by site and context. However, our process and outcomes, undergirded by a broad and systematic needs assessment, offer a generalizable strategy to develop and meet core competencies in GMH training for specialists.

## Disclosures

On behalf of all authors, the corresponding author states there is no conflict of interest. All authors had access to the data, contributed to conducting the study, participating in writing the manuscript, and agree with the results.
